# A Possible Cause of Azoturia

**Published:** 1900-12

**Authors:** A. W. Balch

**Affiliations:** Laboratory of Pharmacology, Harvard Medical School


### A Possible Cause of Azoturia.

A. W. BALCH.

(*Laboratory of Pharmacology, Harvard Medical School.*)

The similarity between certain of the symptoms existing in the disease azoturia and those commonly found in lead-poisoning seemed to make the chemical examination of urines and tissues in these cases desirable.

Examination of the urines from both azoturia cases and normal horses showed the presence of lead in several instances, although negative results were also obtained in both the normal and pathological cases. Chemical examinations were then made of brain, liver, spleen, kidney, and psoas muscle whenever possible, in order that the distribution of lead in the system might be studied.

Organs were taken from normal horses and those in which death had followed azoturia, pneumonia, and colic. As in the urines, lead was found in the tissues of cases other than azoturia, but the amounts were much larger and the lead was much more widely distributed in the azoturia cases.

The quantity found in several of these cases, notably the livers, was very large, indeed, as shown approximately by the final tests employed, while in cases other than azoturia a large amount was never found.

The qualitative results have been placed in tabular form, but the quantities could not be expressed, as we have no method for accurately determining these small amounts of lead.

Organs from Azoturia Cases.

| No. | Brain. | Liver. | Spleen. | Kidney. | Psoas. | Urine. | Blood. | Remarks. |
|---|---|---|---|---|---|---|---|---|
| 12 | + | + | + | + | + | — | | |
| 17 | | + | | — | | + | | |
| 22 | + | + | + | + | + | + | ?— | Elimination of lead increased by potassium iodide. |
| 23 | + | + | | — | | — | | Lead found in urine after potassium iodide. |
| 26 | + | + | + | + | — | | + | |
| 33 | | + | + | + | + | | | |
| 35 | + | — | + | — | + | — | + | |
| 37 | + | + | + | + | + | | + | |

Organs from Cases other than Azoturia.

| No. | Brain. | Liver. | Spleen. | Kidney. | Psoas. | Urine. | Blood. | Remarks. |
|---|---|---|---|---|---|---|---|---|
| 25 | | + | — | + | + | — | | Pneumonia. |
| 27 | | — | — | — | — | | | Runaway. |
| 28 | + | + | — | — | + | | — | Some interstitial change in kidney; broken leg. |
| 34 | | + | — | — | + | | | Colic. |
| 36 | — | — | — | — | ?+ | | | Colic. |
| 38 | — | + | — | + | — | | | Colic. |

| No. | Urines. Azoturia. | No. | Urines. Azoturia. | No. | Urines. Azoturia. |
|---|---|---|---|---|---|
| 1 | + | 7 | + | 22 | +[1] |
| 2 | + | 12 | — | 23 | —[2] |
| 3 | + | 15 | — | 24 | — |
| 4 | + | 16 | + | 29 | — |
| 5 | — | 17 | + | 30 | — |
| 6 | + | 21 | + | 31 | — |

| No. | Urines. Normal Horses. | No. | Urines. Normal Horses. | No. | Urines. Normal Horses. |
|---|---|---|---|---|---|
| 8 | — | 11 | — | 25 | — |
| 9 | + | 13 | + | 28 | — |
| 10 | — | 14 | + | 32 | — |

The results of chemical examination of the spleen are particularly noticeable.

In every case of azoturia lead was found in the spleen, while it was found in none of the other cases examined.

Potassium iodide was administered in two cases of azoturia, numbered in our tables 22 and 23.

In Case 23 no lead was found before the administration of potassium iodide, but after its use very large tests were obtained.

In Case 22 the amount eliminated was greatly increased.

Lack of material prevented similar experiments in normal horses. For examination of urine about one litre was used in each case. For tissue examination one-half the brain, weighing when fresh about 300 grammes, or dried, 50 grammes, and of other tissues 300 grammes fresh or 100 grammes dry, were used. The tissue or dry urine was treated with an excess of nitric acid and allowed to stand until the violent reaction was over.

To the resulting fluid, after concentration to a small bulk, 28 c.c. concentrated sulphuric acid was added and the heat continued until charring resulted. The fat was not removed. The process was then continued by alternately adding nitric acid and charring by heat until the organic matter was practically destroyed. Dur-

[1] Increased by potassium iodide.

[2] Found after potassium iodide administration.

ing the heating a part of the sulphuric acid was always decomposed so that the product (free from nitric acid) varied in bulk from 10 c.c. to 25 c.c., depending in part upon the amount of solids present.

This product was diluted with an equal volume of water and the mixture again diluted with an amount of alcohol equal in volume to the sulphuric acid and water used. From this mixture after standing several days the lead was deposited as sulphate. The deposit was washed on a paper filter with 50 per cent. alcohol made acid with sulphuric acid, and was then extracted with 10 per cent. ammonium acetate solution. From this solution the lead was precipitated as sulphide, and in case much iron was present it was removed with very dilute hydrochloric acid. The lead was then converted into chloride by concentrated hydrochloric acid, and removed from the filter by hot water. After evaporation the lead chloride was dissolved in a few drops of hot water, the solution filtered if necessary, and four final tests made—*i.e.*, potassium dichromate, potassium iodide, sulphuric acid, and hydrogen sulphide. Lead was considered as present only when all four tests were obtained.

The paper is presented merely as a preliminary statement of the facts obtained thus far, and in no sense as conclusive evidence of the origin of azoturia. The wide distribution of lead makes proof by chemical means alone impossible, but the results obtained are certainly suggestive.

An attempt is to be made to produce the disease in normal horses by the administration of lead salts.

